# A quadruple mutant of Arabidopsis reveals a β-carotene hydroxylation activity for LUT1/CYP97C1 and a regulatory role of xanthophylls on determination of the PSI/PSII ratio

**DOI:** 10.1186/1471-2229-12-50

**Published:** 2012-04-18

**Authors:** Alessia Fiore, Luca Dall'Osto, Stefano Cazzaniga, Gianfranco Diretto, Giovanni Giuliano, Roberto Bassi

**Affiliations:** 1 Italian National Agency for New Technologies, Energy and Sustainable Development (ENEA), Casaccia Research Center, Via Anguillarese 301, 00123 Rome, Italy; 2 Dipartimento di Biotecnologie, Università di Verona, Strada Le Grazie 15, 37134 Verona, Italy; 3 ICG-3: Phytosphäre Forschungszentrum Jülich, 52425 Jülich, Germany; 4 Dipartimento di Biotecnologie, Università di Verona, Strada Le Grazie 15, 37134 Verona, Italy

## Abstract

**Background:**

Xanthophylls are oxygenated carotenoids playing an essential role as structural components of the photosynthetic apparatus. Xanthophylls contribute to the assembly and stability of light-harvesting complex, to light absorbance and to photoprotection. The first step in xanthophyll biosynthesis from α- and β-carotene is the hydroxylation of ε- and β-rings, performed by both non-heme iron oxygenases (CHY1, CHY2) and P450 cytochromes (LUT1/CYP97C1, LUT5/CYP97A3). The Arabidopsis triple *chy1chy2lut5 *mutant is almost completely depleted in β-xanthophylls.

**Results:**

Here we report on the quadruple *chy1chy2lut2lut5 *mutant, additionally carrying the *lut2 *mutation (affecting lycopene ε-cyclase). This genotype lacks lutein and yet it shows a compensatory increase in β-xanthophylls with respect to *chy1chy2lut5 *mutant. Mutant plants show an even stronger photosensitivity than *chy1chy2lut5*, a complete lack of qE, the rapidly reversible component of non-photochemical quenching, and a peculiar organization of the pigment binding complexes into thylakoids. Biochemical analysis reveals that the *chy1chy2lut2lut5 *mutant is depleted in Lhcb subunits and is specifically affected in Photosystem I function, showing a deficiency in PSI-LHCI supercomplexes. Moreover, by analyzing a series of single, double, triple and quadruple Arabidopsis mutants in xanthophyll biosynthesis, we show a hitherto undescribed correlation between xanthophyll levels and the PSI-PSII ratio. The decrease in the xanthophyll/carotenoid ratio causes a proportional decrease in the LHCII and PSI core levels with respect to PSII.

**Conclusions:**

The physiological and biochemical phenotype of the *chy1chy2lut2lut5 *mutant shows that (i) LUT1/CYP97C1 protein reveals a major β-carotene hydroxylase activity *in vivo *when depleted in its preferred substrate α-carotene; (ii) xanthophylls are needed for normal level of Photosystem I and LHCII accumulation.

## Background

Carotenoids are a group of C_40 _pigments that contain a conjugated double-bond system, leading to strong absorption of visible light and antioxidant properties. They are widely distributed among taxa, ranging from cyanobacteria and fungi to red and green algae and land plants [1]. Xanthophylls are oxygenated carotenoids that play a crucial role in the photosynthetic apparatus of higher plants [2]. Their composition in plants is remarkably conserved and consists of five major xanthophylls, the most abundant being the β-ε-xanthophyll lutein, and the four β-β-xanthophylls violaxanthin, neoxanthin, antheraxanthin and zeaxanthin [3]. Xanthophylls act both as photoreceptors, absorbing light energy which is used in photosynthetic electron transport, and as photoprotectants of the photosynthetic apparatus from excess light and from the reactive oxygen species (ROS) generated during photosynthesis [4-7]. Moreover, they are structural elements of the photosynthetic apparatus: LHCII, the major light-harvesting complex (LHC) of Photosystem (PS) II, binds lutein, violaxanthin and neoxanthin at four distinct binding sites called respectively L1, L2, N1 and V1 [8]; the occupancy of L1 site was shown to be essential for protein folding [9].

**Figure 1:**
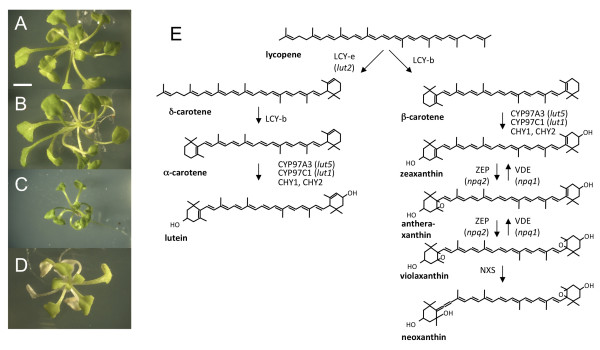
**Growth and morphology of wild-type and carotenoid biosynthesis mutant plants**. Phenotypes of 6-weeks-old wild type (**A**), *lut2 *(**B**), *chy1chy2lut5 *(**C**), and *chy1chy2lut2lut5 *(**D**) plantlets, grown at 30 μmol photons m^-2 ^s^-1^. (**E**) Biosynthetic pathway of carotenoids in *A. thaliana; *names of the enzymes controlling each step are indicated: lycopene ε-cyclase (LCY-ε); lycopene β-cyclase (LCY-β); β-carotene hydroxylase (CYP97A3); ε-β-carotene hydroxylase (CYP97C1); β-carotene hydroxylase 1 and 2 (CHY1, CHY2); zeaxanthin epoxidase (ZEP); violaxanthin deepoxidase (VDE); neoxanthin synthase (NXS). Names of Arabidopsis knock-out mutants are indicated in parentheses. Scale bar = 5 mm.

Xanthophyll biosynthesis in plants is divided in two distinct branches: the α branch leads to the formation of the ε-β-hydroxylated xanthophyll lutein from α-carotene, while the β branch leads to the production of β-β-hydroxylated xanthophylls (zeaxanthin, antheraxanthin, violaxanthin and neoxanthin) from β-carotene (Figure 1E). Recent studies on carotenoid biosynthetic mutants of *Arabidopsis thaliana *have improved our understanding on xanthophyll accumulation at the molecular level. The first steps in plant xanthophyll biosynthesis are the hydroxylation of α- and β-carotene. Two different classes of enzymes are involved: the ferredoxin-dependent di-iron oxygenases (CHY1 and CHY2) which are active in β-ring hydroxylation, and the cytochromes P450 (LUT1/CYP97C1, LUT5/CYP97A3) [10-14] which are active in hydroxylation of both the ε-ring and β-ring of α-carotene, although the activity of LUT5 on ε-rings is low [13]. It has been suggested that a third chloroplast-targeted member of the CYP97 family, CYP97B3 might have a role in carotenoid biosynthesis [14]. This hypothesis is however in contrast with the complete lack of xanthophylls in the quadruple *chy1chy2lut1lut5 *mutant [14], suggesting that CHY1, CHY2, LUT1/CYP97C1 and LUT5/CYP97A3 are the complete complement of carotene hydroxylases in *A. thaliana*.

The strong phenotypes of mutants with altered xanthophyll composition imply that the presence and relative amounts of these pigments have a key role for plant fitness. The *lut2 *mutant, affected in ε-ring formation, lacks lutein [15] and shows a complex phenotype with reduced antenna size, photodamage in high light (HL) due to impaired chlorophyll triplet quenching [16] and decrease of non-photochemical quenching (NPQ) [17]. Additional features include over-accumulation of zeaxanthin in HL with respect to wild-type and monomerization of LHCII. Lack of both lutein and zeaxanthin further decreases the capacity for photoprotection in both Arabidopsis and Chlamydomonas [16,18-20]. The double *chy1chy2 *mutant, in which the two non-heme β-hydroxylases are deleted, shows reduced levels of β-β-xanthophylls and decreased resistance to photooxidation [10]. Introduction of the *lut5 *mutation in the *chy1chy2 *background leads to the almost complete disappearance of β-xanthophylls and strong photosensitivity [6,12,14]. Neoxanthin preserves PSII from photoinactivation by superoxide anions [21] while violaxanthin and zeaxanthin show enhanced activity in singlet oxygen scavenging [6]. In order to further detail the effects of altered xanthophyll composition on the organization of photosynthetic complexes and gain understanding on the regulatory events controlling xanthophyll biosynthesis in Arabidopsis, we have introduced the *lut2 *mutation in the semi-lethal *chy1chy2lut5 *background. Surprisingly, the *chy1chy2lut2lut5 *mutant shows increased presence of β-β-xanthophylls with respect to *chy1chy2lut5*. The PSI/PSII ratio in this mutant is severely decreased as well as the level of total xanthophyll accumulation, suggesting that the latter have a key role, beside photoprotection, in regulating photosystem stoichiometry.

## Results

### Construction of the chy1chy2lut2lut5 quadruple mutant

To generate the quadruple mutant *chy1chy2lut2lut5*, the homozygous triple mutant *chy1chy2lut2 *was crossed with the homozygous single mutant *lut5 *[12]. All T-DNA insertions were in the Columbia background and appropriate oligonucleotides were used to confirm the presence of the insertions and their homozygous *vs*. heterozygous state (Additional file 1: Figure S1 and Additional file 1: Table S1) [12]. The mutant was maintained as a triple homozygous, single heterozygous stock: two different parental genotypes were used, heterozygous for either *LUT5/CYP97A3 *or *CHY2*, with similar results (Additional file 1: Figure S2). The *lut2 *and *chy1chy2lut5 *lines were included in this characterization, representing respectively the lutein-less and β-xanthophyll-less controls. When selfed, the progeny of each single heterozygous stock, as well as wild-type, *chy1chy2lut5 *and *lut2 *seeds, were grown under low light conditions (30 μmol photons m^-2 ^s^-1^) both in agar plates containing sucrose (see Methods) and in soil. No quadruple homozygous mutants were recovered in soil after 1 week of growth, while the progeny segregated in a 1:3 ratio for white: green seedlings in agar plates. Wild type and *lut2 *plants did not display a visible phenotype after 6 weeks of growth in agar plates, whereas mutants *chy1chy2lut5 *and *chy1chy2lut2lut5 *showed, respectively, reduced growth and paler leaves (Figure 1A-D).

### Pigment composition

We analyzed by HPLC-DAD-MS the pigment content of six-week-old leaves of wild type, *lut2, chy1chy2lut5 *and *chy1chy2lut2lut5 *plants grown on agar plates (Tables 1, 2 and Additional file 1: Table S2). Pigments were resolved on a C-30 column, able to separate *cis*- from *trans*-carotenoids and their identity was confirmed by co-migration with authentic standards and high resolution MS (Additional file 1: Table S2). *chy1chy2lut5 *and *chy1chy2lut2lut5 *plants showed an increase in chlorophyll a/b ratio, as well as a reduced chlorophyll (Chl) and carotenoids (Car) content per fresh weight, with respect to both wild-type and *lut2*; the effects were more severe in the quadruple mutant, that showed a significant reduction of Chl/Car ratio (2.1) with respect to the other genotypes (~3.3, Table 1). Wild-type leaves accumulate four major carotenoids (neoxanthin, violaxanthin, lutein and β-carotene) and trace amounts of α-carotene. Mutants show distinct composition of the xanthophyll fractions: lutein represents > 98% of total xanthophylls in *chy1chy2lut5 *plants; *lut2 *and *chy1chy2lut2lut5 *do not contain lutein and accumulate violaxanthin, antheraxanthin, zeaxanthin and neoxanthin; *chy1chy2lut2lut5 *shows a higher content of β-β-xanthophylls (24% of total carotenoids) with respect to *chy1chy2lut5 *(0.7% of total carotenoids). β-carotene content is strongly increased in *chy1chy2lut2lut5 *with respect to the wild-type and the other mutants (Table 2). As a result, the xanthophyll/carotene ratio changes dramatically, ranging from 2.5 ± 0.7 in wild-type to 0.3 ± 0.1 in *chy1chy2lut2lut5*.

**Table 1:** Pigment content of leaf tissue from wild-type and mutant genotypes

	chl a/b	chl/car	Chl content (μg/g FW)	Car content (μg/g FW)
**WT**	3.2 ± 0.5^a, b^	3.3 ± 0.8^a, b^	801 ± 88^a^	244 ± 51^a^
***lut2***	3.2 ± 0.2^a^	3.3 ± 0.7^a, b^	742 ± 79^a^	222 ± 38^a^
***chy1chy2lut5***	3.9 ± 0.3^b^	3.4 ± 0.7^a^	569 ± 76^b^	166 ± 25^b^
***chy1chy2lut2lut5***	7.9 ± 0.3^c^	2.1 ± 0.4^b^	303 ± 42^c^	146 ± 21^b^

Pigment composition was quantified via LC-DAD-MS in dark-adapted leaves from 6-weeks-old plants. Data are expressed as mean ± SD (n = 4). FW, fresh weight. Values marked with the same letters are not significantly different from each other within a column (*P *> 0.05).

**Table 2:** HPLC analysis of leaf carotenoid content (μg/g FW) in dark-adapted plants

	Carotenoid content (μg/g FW)								
	**neoxanthin**	**violaxanthin**	**antheraxanthin**	**lutein**	**zeaxanthin**	**α-carotene**	**β-carotene**	**β-β-xantophylls**	**xanthophylls/carotenes**
**WT**	18.6 ± 1.6^a^	44.1 ± 5.8^a^	nd	105.6 ± 22.4^a^	nd	4.4 ± 0.6^a^	62.1 ± 14.8^a^	62.7 ± 6.0^a^	2.5 ± 0.7^a^
***lut2***	22.1 ± 0.7^b^	94.7 ± 4.9^b^	14.6 ± 1.4^a^	nd	5.0 ± 0.6^a^	nd	74.9 ± 5.1^a^	136.4 ± 5.1^b^	1.8 ± 0.1^a^
***chy1chy2lut5***	0.3 ± 0.2^c^	0.9 ± 0.6^c^	nd	69.1 ± 4.7^b^	nd	51.0 ± 2.7^b^	42.8 ± 1.4^b^	1.2 ± 0.6^c^	0.7 ± 0.1^b^
***chy1chy2lut2lut5***	8.4 ± 0.1^d^	13.6 ± 0.2^d^	8.5 ± 0.1^b^	nd	3.4 ± 0.1^b^	nd	109.4 ± 2.3^c^	33.9 ± 0.3^d^	0.3 ± 0.1^c^

Plants grown at 30 μmol photons m^-2 ^s^-1 ^for 6 weeks were dark-adapted, then carotenoids were extracted and quantified via LC-DAD-MS. Data are expressed as mean ± SD (n = 4). nd, not detectable; FW, fresh weight. Values marked with the same letters are not significantly different from each other within a column (*P *> 0.05).

β-carotene accumulation is expected in *chy1chy2lut2lut5*, in which the biosynthetic flux is diverted towards the β-β-branch by the lack of three out of four β-carotene hydroxylases (CHY1, CHY2 and CYP97A3/LUT5) (Figure 1E); however, β-β-xanthophyll accumulation in this mutant suggests that the fourth hydroxylase (CYP97C1/LUT1) is more active toward β-carotene in this background than in the *chy1chy2lut5 *parent, resulting in 28-fold higher levels of β-β-xanthophylls.

### Gene expression

**Figure 2:**
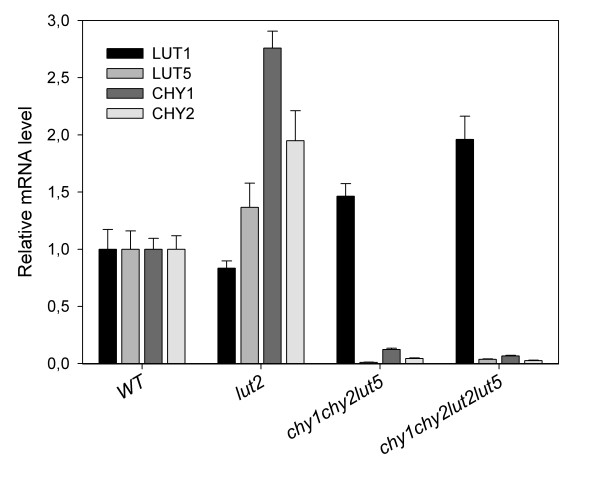
**Real-time PCR expression profile of Arabidopsis carotenoid hydroxylase genes**. For individual genes, the relative mRNA levels were normalized with respect to the *TUBULIN *housekeeping transcript and then to wild-type levels. RNA was extracted from dark-adapted, 6-weeks-old plants (see Methods for details). Data are expressed as mean ± SD (n = 3).

We measured the *LUT1, LUT5, CHY1 and CHY2 *mRNA levels by real time PCR in the different mutants (Figure 2). All mRNAs are almost completely absent in the corresponding mutants; *LUT5, CHY1 and CHY2 *are induced in the *lut2 *mutant, that accumulates higher levels of β-carotene; *LUT1 *is induced in the *chy1chy2lut5 *and, even more, in the *chy1chy2lut2lut5 *mutant, which shows drastically reduced xanthophyll/carotene ratios; however, the increase of *LUT1 *levels between the two mutants is only 1.3-fold, while the increase of β-β-xanthophylls is 28-fold.

### Photosynthesis-related functions: PSII quantum efficiency and non-photochemical quenching of chlorophyll fluorescence

The impact of xanthophyll depletion on photosynthesis was investigated by room temperature chlorophyll fluorescence measurements (Table 3). The variable/maximum fluorescence yield (F_v_/F_m_) of dark-adapted leaves reflects changes in PSII photochemical efficiency [22]. *lut2 *had the same F_v_/F_m _ratio as wild-type (0.80), while the triple *chy1chy2lut5 *and quadruple *chy1chy2lut2lut5 *mutants scored, respectively, values of 0.68 and 0.51 (Table 3). The efficiency of PSII photochemistry (Φ_PSII_) gives a measure of the rate of linear electron transport, an indication of the photosynthetic activity [23]. Chlorophyll fluorometry revealed a significant reduction in Φ_PSII _in both *chy1chy2lut5 *and *chy1chy2lut2lut5 *(0.07 and 0.08 respectively, with respect to 0.17 in wild-type plants, Table 3), confirming that efficient light use is compromised by β-β-xanthophyll depletion.

**Table 3:** Analysis of room temperature chlorophyll fluorescence during steady-state photosynthesis

	Fv/Fm	ΦPSII
**WT**	0.81 ± 0.03^a^	0.17 ± 0.05^a^
***lut2***	0.80 ± 0.02^a^	0.12 ± 0.01^a, b^
***chy1chy2lut5***	0.68 ± 0.02^b^	0.07 ± 0.01^c^
***chy1chy2lut2lut5***	0.51 ± 0.10^c^	0.08 ± 0.05^b, c^

Detached leaves of wild-type and mutant plants grown at 30 μmol photons m^-2 ^s^-1 ^for 6 weeks were given 20 min of illumination (1000 μmol photons m^-2 ^s^-1^), then photosynthetic parameters were provided by analysis of RT chlorophyll fluorescence: maximum quantum yield of PSII (F_v_/F_m_) and efficiency of PSII photochemistry (Φ_PSII_). Data are expressed as mean ± SD (n = 4). Values marked with the same letters are not significantly different from each other within a column (*P *> 0.05).

**Figure 3:**
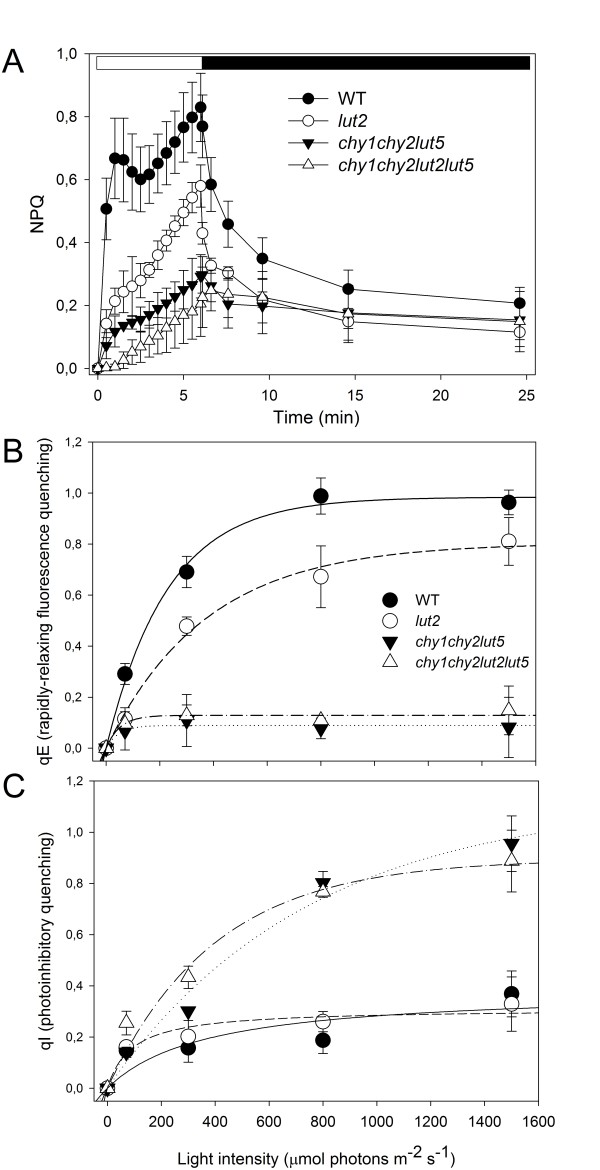
**NPQ analysis of wild-type and mutant genotypes**. (**A**) Kinetics of NPQ induction and relaxation were recorded with a pulse amplitude modulated fluorometer. Chlorophyll fluorescence was measured in dark-adapted leaves, during 7 min of illumination at 1000 μmol photons m^-2 ^s^-1 ^followed by 18 min of dark relaxation. (**B**) Amplitude of the reversible energy dissipation (qE). (**B, C**) Extent of feed-back de-excitation (qE, panel B) and photoinhibitory quenching (qI, panel C) determined at a series of irradiances as a difference between NPQ values upon illumination and following 15 min dark relaxation. Symbols and error bars show means ± SD (n = 4).

Non-photochemical quenching (NPQ) of chlorophyll fluorescence is the fastest photoprotective mechanism in the chloroplast: thermal dissipation is activated within few seconds upon exposure to excess light and it protects photosynthesis by decreasing the lifetime of singlet chlorophylls [24] in order to minimize generation of ROS in the PSII [4]. NPQ was measured on detached leaves, in saturating CO_2 _(Figure 3). Wild-type plants, upon short illumination at saturating light intensity (1000 μmol photons m^-2 ^s^-1^, 7 min), showed a rapid rise of NPQ, reaching a maximum value of 0.8. Most of this NPQ relaxed rapidly in the dark, thus reflecting the ΔpH-dependent de-excitation of excess energy measured as qE, the rapidly-reversible component of NPQ. *lut2 *showed NPQ kinetics in agreement with published results [6,19], with lower amplitude and slower rise than wild-type plants (Figure 3A). The other mutants showed a strong reduction in NPQ, scoring 0.30 in *chy1chy2lut5 *and 0.22 in *chy1chy2lut2lut5; *furthermore, upon correction for residual quenching after dark relaxation (photoinhibitory quenching, qI), both mutants showed very little recovery (Figure 3A), suggesting that the measured fluorescence quenching was mainly due to photoinhibition, and the capacity for qE was strongly reduced in mutant leaves (Figure 3A). Furthermore, NPQ kinetics were measured during steady-state photosynthesis, upon a prolonged illumination with increasing light intensities (ranging from 70 to 1500 μmol photons m^-2 ^s^-1^, 20 min). All genotypes showed chlorophyll fluorescence quenching, whose magnitude increased with irradiance. However, fluorescence quenching in wild-type and *lut2 *leaves was mainly due to the qE-type of NPQ (Figure 3B), while in both *chy1chy2lut5 *and *chy1chy2lut2lut5 *the main component of NPQ was qI-type, irreversible quenching (Figure 3C). These data confirm that both the reduction of β-β-xanthophylls and the lack of lutein are responsible for impaired NPQ kinetics.

### Photosensitivity under short-term stress conditions

When photosynthetic organisms are exposed to light in excess, photo-oxidative stress occurs within the chloroplast, with production of ROS such as singlet oxygen (^1^O_2_), leading to oxidative damages to a large variety of biomolecules.

**Figure 4:**
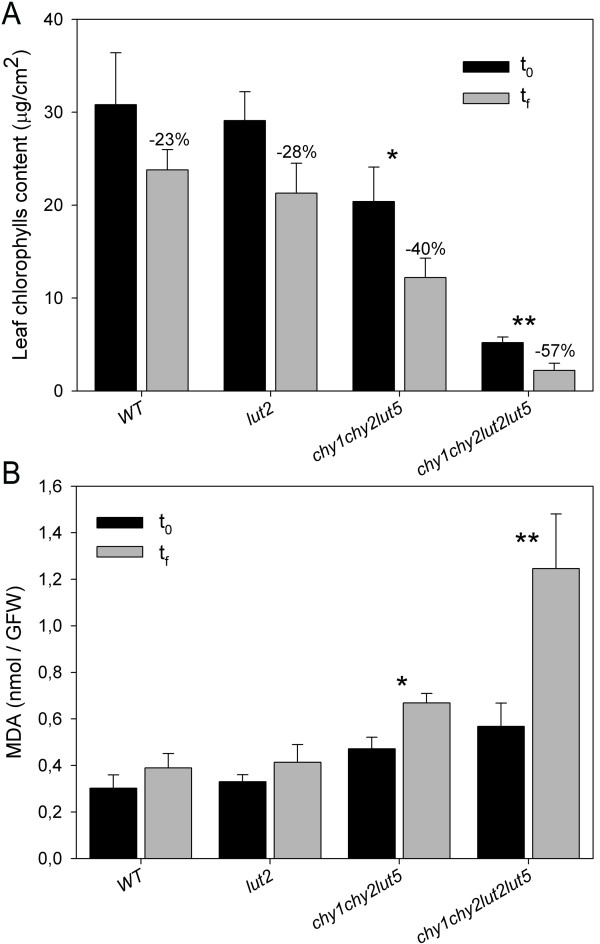
**Photo-oxidation of Arabidopsis wild-type and mutant genotypes under photoxidative stress**. Detached leaves on wet paper were treated at 900 μmol photons m^-2 ^s^-1 ^at 5°C for 3.5 h, then chlorophyll bleaching (**A**) and MDA formation (**B**) were recorded. Data are expressed as means ± SD (n = 4). Significant difference (*P *< 0.05 (*) or *P *< 0.02 (**)) against wild type within each treatment are marked. Chlorophyll and MDA contents were quantified before (t_0_) or after (t_f_) the high-light stress.

The β-xanthophyll-depleted mutants *chy1chy2lut5 *and *chy1chy2lut2lut5*, even upon growth in low light (30 μmol photons m^-2 ^s^-1^), showed signs of photooxidation: lower chlorophyll content and PSII quantum yield, retarded growth and paler leaves with respect to wild-type and *lut2 *plants (Tables 1, 3). Therefore, in order to assess whether lower xanthophyll levels affect the capacity to prevent chloroplast photooxidation, leaves from wild-type and mutant plants grown in low light were transferred to strong light (900 μmol photons m^-2 ^s^-1^) and low temperature (5°C) for 3.5 h; the combination of low temperature and high light intensity is known to enhance the induction of both PSII photoinhibition and membrane photooxidation in leaves, since the enzymes of the Calvin cycle are slowed down and the light harvested by photosystems rapidly exceed the capacity of plants to use this energy. Thus, the treatment produces a photooxidative stress, which can be measured as a decrease in the chlorophyll content and an increase in oxidation of membrane lipids. HL treatment was effective in producing higher pigment bleaching in *chy1chy2lut5 *(40% reduction) and *chy1chy2lut2lut5 *(57% reduction), while wild-type and *lut2 *leaves were less affected, loosing around 25% of their chlorophyll content (Figure 4A). To investigate the level of membrane lipid peroxidation, the same leaves were analyzed for MDA content (malondialdehyde, a byproduct of lipid peroxidation): *chy1chy2lut2lut5 *and *chy1chy2lut5 *leaves showed higher accumulation of MDA upon stress treatment (+120% and +45%, respectively), thus a far higher level of lipid peroxidation with respect to wild-type and *lut2 *plants (+25%); *chy1chy2lut2lut5 *plants showed a far higher photosensitivity in high-light than *chy1chy2lut5 *(Figure 4B); the latter was the xanthophyll mutant with the highest light sensitivity described so far [6]. Results clearly show an unprecedented level of photosensitivity in *chy1chy2lut2lut5 *plants, thus implying a severe impairment of the photoprotection mechanisms in this xanthophyll-depleted mutant.

### Organization and stoichiometry of pigment binding complexes

**Figure 5:**
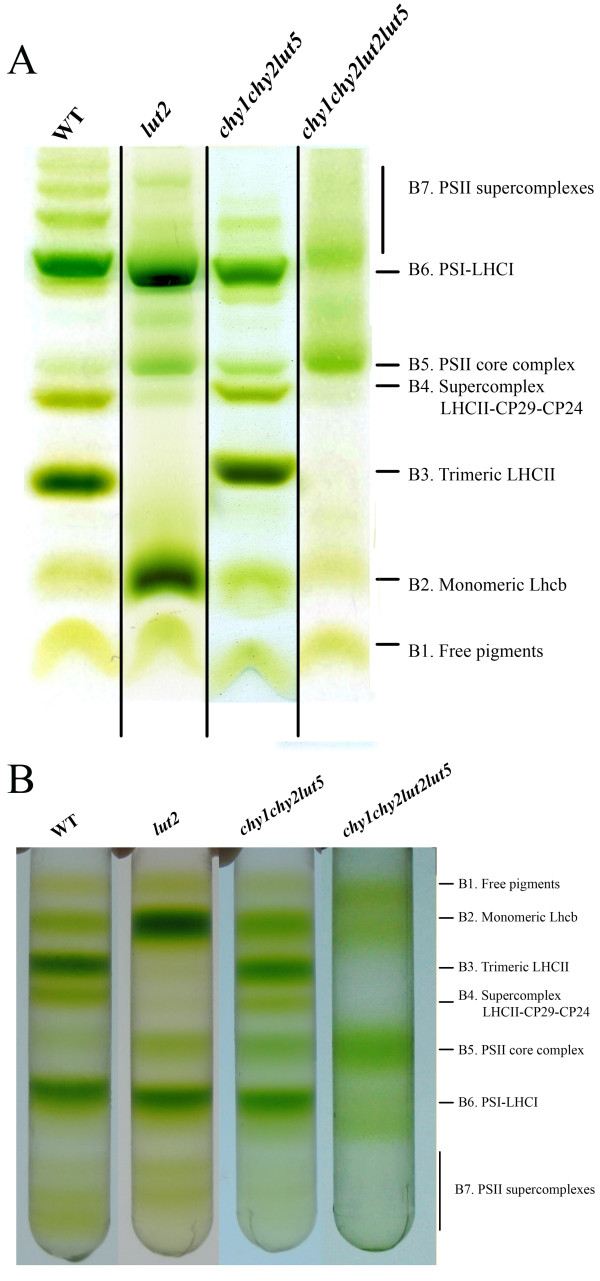
**Analysis of pigment-protein complexes of wild-type and mutants**. (**A**) Thylakoid pigmented complexes were separated by non-denaturing Deriphat-PAGE (**A**) or by sucrose density gradient fractionation (**B**) upon solubilization with 0.8% α-DM.

**Figure 6:**
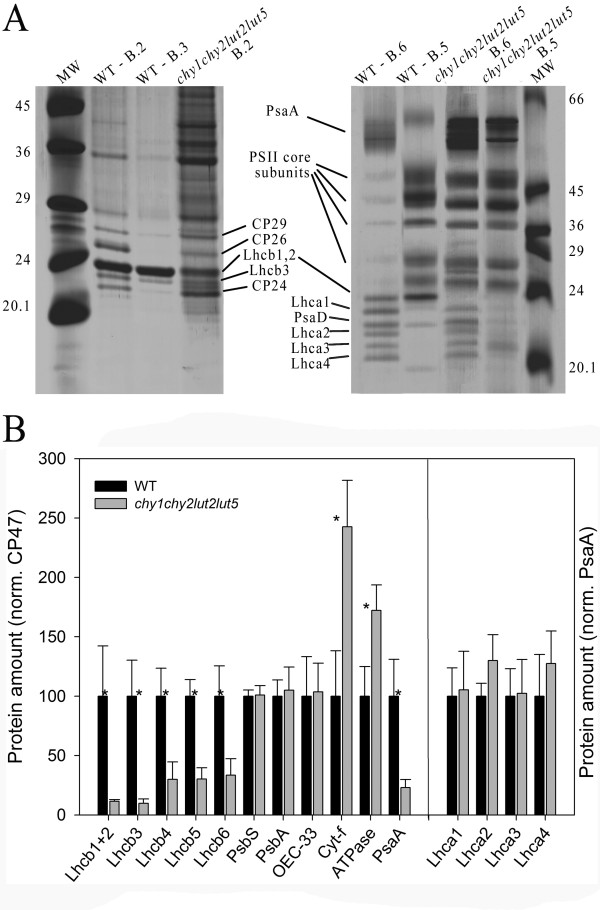
**Analysis of polypeptide composition of wild-type and *chy1chy2lut2lut5 *thylakoid membrane**. (**A**) Pigment-protein complexes isolated by sucrose density gradient fractionation (Figure 5B) were analyzed by Tris-tricine (*left panel*) of Tris-glycine (*right panel*) SDS-PAGE. Main protein components of each fraction are indicated. MW, molecular weight marker. (**B**) Results of the immuno-titration of thylakoid proteins. Immunoblot analyses were performed with antibodies directed against individual gene products. Data of PSII subunits were normalized to the PsbB content, that of Lhca to the PsaB content, and expressed as a percentage of the corresponding wild-type content. Significantly different values in protein amount than the corresponding wild-type, according to Student's *t *test (*P *< 0.05), are marked (*).

The extreme sensitivity to photo-oxidative stress of the *chy1chy2lut2lut5 *mutant could be due to altered pigment composition, to altered protein composition of photosystems, or to both. This mutant showed the highest Chl *a/b *ratio, and the lowest chlorophyll content and xanthophyll/carotene ratio of all analyzed genotypes (Tables 1, 2). Since both Chl *b *and xanthophylls are associated with LHC, their decrease suggests a decrease in antenna size. We investigated the organization of pigment-protein complexes in thylakoids by non-denaturing Deriphat-PAGE and by sucrose density gradient fractionation of solubilized thylakoids, followed by SDS-PAGE of the fractions (Figures 5, 6). Seven major green bands were resolved upon solubilization of wild-type thylakoid membranes with 0.8% dodecyl-α-D-maltoside (α-DM) on Deriphat-PAGE [25]. The PSI-LHCI complex was the major band (B6) in the upper part of the gel, while the components of the PSII-LHCII complex migrated as multiple bands, namely the PSII core (B5) and the antenna moieties, including the CP29-CP24-LHCII-M supercomplex (B4) [26], LHCII trimer (B3) and monomeric Lhcb (B2). Bands with high apparent masses were detected in the upper part of the gel (B7) containing non-dissociated PSII supercomplexes.

The *chy1chy2lut5 *pattern was very similar to that of wild-type, showing no major qualitative changes in the organization of the photosynthetic apparatus: the main differences consisted in a higher PSII core/Lhcb ratio and a lower content in PSII supercomplexes (B7) [6]. In the genotypes lacking lutein, namely *lut2 *and *chy1chy2lut2lut5*, the trimeric organization of LHCII was disrupted, as previously described [17]. However, thylakoid membranes isolated from *chy1chy2lut2lut5 *plants showed additional features, namely the complete absence of bands 3, 4 and 7; bands 2 and 6 were much less represented than in wild-type, while PSII core complex was the most abundant among pigment-protein complexes (Figure 5A).

In order to obtain sufficient amounts of pigment-protein complexes for further analysis, solubilized thylakoids from wild type and mutants were fractionated by sucrose gradient ultracentrifugation (Figure 5B); the results confirmed that trimeric LHCII band, as well as the CP29-CP24-LHCII supercomplex completely disappeared in the *chy1chy2lut2lut5 *mutant; further differences consisted into reduced levels of monomeric Lhcb (band 2) and a much higher PSII/PSI ratio (band 5 *vs*. band 6) with respect to the other genotypes. The reduction of monomeric Lhcbs in the *chy1chy2lut2lut5 *mutant occurs in spite of the presence of the *lut2 *mutation, which favors LHCII monomerization (compare band 2 in wild type *vs*. *lut2 *and in *chy1chy2lut5 vs*. *chy1chy2lut2lut5*, Figure 5B).

Fractions collected from the sucrose gradients of wild-type and *chy1chy2lut2lut5 *(bands 2-6) were further characterized by SDS-PAGE using two buffer systems (Figure 6A) and by absorption spectroscopy (Additional file 1: Figure S3). According to previous results with α-DM, SDS-PAGE analyses of wild-type fractions showed that band 2 contained the minor antennae CP29, CP26 and CP24 as well as components of monomerized LHCII, while band 3 contained Lhcb1-3 polypeptides only (Figure 6A, left panel) [27]. Band 5 was enriched in PSII core complex (Figure 6A, right panel) [28], nevertheless the Chl *b *absorption (Additional file 1: Figure S3) suggests it retains Lhcb proteins; band 6 contained almost exclusively the PSI-LHCI complex (Figure 6A, right panel and Additional file 1: Figure S3) [29]. Band 2 from *chy1chy2lut2lut5 *contained the same polypeptides as the corresponding band from wild-type, although the relative amounts of the Lhcb1-3 polypeptides and CP26 were decreased (Figure 6A, left panel). The data confirm that LHCII is present in the mutant, however in far lower amounts than in wild-type, and is in its monomeric aggregation state. Band 5 from the mutant contained almost exclusively PSII core complex polypeptides (Figure 6A and Additional file 1: Figure S3), band 6 from the mutant contained PSI-LHCI.

The levels of selected proteins in wild-type and *chy1chy2lut2lut5 *thylakoids were determined by quantitative western-blot analysis using PsbB (CP47) as internal control (Figure 6B): all Lhcb subunits were reduced in *chy1chy2lut2lut5 *with respect to wild-type thylakoids; PsbA (D1), OEC-33 and PsbS, subunits of the PSII core complex, were present in the same level in both genotypes, while cytochrome *f *and ATPase β-subunit were in higher amounts in *chy1chy2lut2lut5*. Immunoblotting using PsaA as internal standard showed that each Lhca protein was present in wild-type amounts, thus suggesting that the Lhca/PSI ratio is conserved in the mutant. In contrast, the PSI/PSII ratio was extremely low in *chy1chy2lut2lut5*, reaching approximately 22% of wild-type value (Figure 6B).

The data shown above indicate that xanthophyll depletion in the *chy1chy2lut2lut5 *plants causes a strong reduction in the amount of Lhcb proteins per PSII reaction center, and has a negative impact on the total amount of the PSI-LHCI supercomplex. The latter result is unexpected, since xanthophylls are mainly bound to Lhc complexes while core complexes of both photosystems only bind carotenes, implying that xanthophyll abundance should not affect their folding or stability.

**Figure 7:**
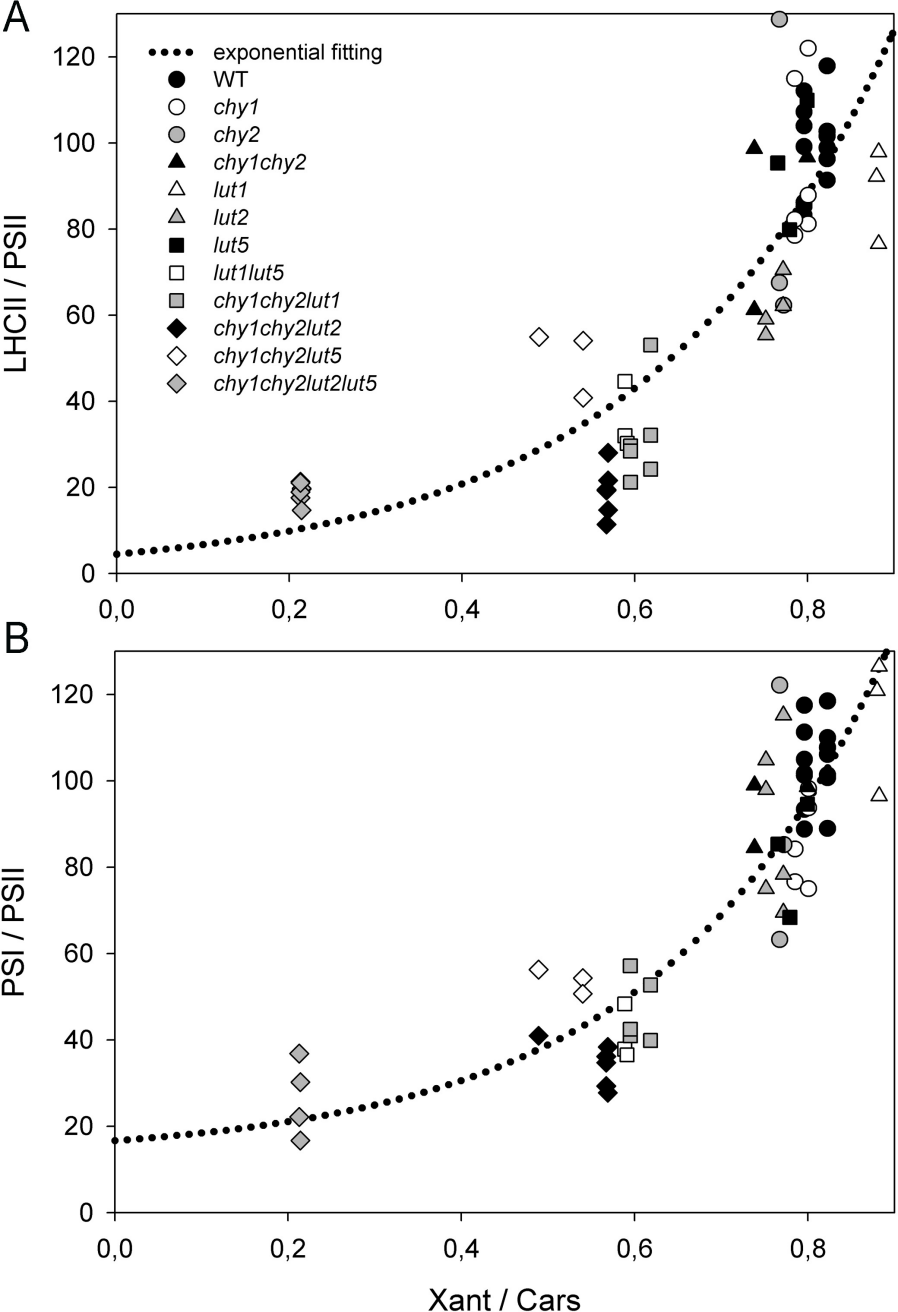
**Relations between LHCII/PSII (A) and PSI/PSII (B) ratios and the relative content of xanthophylls on thylakoids**. Parameters were measured for wild-type and 11 xanthophyll biosynthesis mutants plants previously described ([12]; present ms). LHCII/PSII and PSI/PSII ratios were determined on thylakoid membranes by quantitative western blot, while xanthophyll/carotenoid ratios were quantified by HPLC. Both distributions were fitted with exponential functions (y = y_o _+ a·e^bx^); R_LHCII/PSII_^2 ^= 0.76; R_PSI/PSII_^2 ^= 0.78.

A key question is whether the lower PSI/PSII ratio found in *chy1chy2lut2lut5 *thylakoids is peculiar to this genotype or is a general consequence of altered xanthophyll content. To answer this question, the abundance of LHCII and PSI core with respect to CP47 was assessed by quantitative immunotitration, in thylakoids isolated from 12 Arabidopsis mutants with altered xanthophyll content [6,12]. Figure 7 shows the distribution of LHCII (A) and PSI core (B) amounts, relative to CP47, in the different mutants with different xanthophyll/carotenoid ratios: both distributions show an increase of LHCII/PSII and PSI/PSII at increasing xanthophyll/carotenoid ratios, and the data can be fitted with an exponential function (y = y_o _+ a·e^bx^). The results display a clear correlation between parameters (R_LHCII/PSII_^2 ^= 0.76; R_PSI/PSII_^2 ^= 0.78). Furthermore, quantitative data for the individual pigment-protein complexes accumulation level per fresh weight (namely PSI core, PSII core and LHCII) were plotted *vs*. xanthophyll/carotenoid content in the different mutants (Additional file 1: Figure S4). The distributions show a lower LHCII content per fresh weight at decreasing xanthophyll/carotenoid ratios (panel C), while PSII core (CP47 subunit) content per fresh weight was essentially unaffected by xanthophylls depletion (panel B). Unlike PSII, the PSI core (PsaA subunit) content decreases at decreasing xanthophyll/carotenoid ratios (panel A). These results suggest that xanthophyll depletion not only causes a marked depletion in LHC proteins, as expected from the xanthophylls being ligands of Lhcs, but also modulates the ratio between PSI/PSII, despite β-carotene rather than xanthophylls is the ligand for PSI and PSII core complexes.

## Discussion

In this work, we analyzed the modifications of the photosynthetic apparatus in the Arabidopsis mutant *chy1chy2lut2lut5*, that accumulates β-xanthophylls despite disruption of the three *chy1, chy2 *and *lut5 *genes encoding carotene hydroxylases. In this mutant the only carotene hydroxylase activity is provided by the LUT1 gene product, which allows for accumulation of only 20% of the wild-type xanthophylls. In these conditions, biogenesis of the photosynthetic apparatus was strongly affected yielding into a reduction of PSII antenna size, a decrease of PSI/PSII ratio and an increased photosensitivity.

### LUT1 as a β-carotene hydroxylase

LUT1 was originally reported to be only gene product required for the ε-ring hydroxylation of α-carotene [11]. The first indication of a possible involvement of LUT1 in β-ring hydroxylation came from the analysis of the *chy1chy2lut5 *triple mutant, which accumulates lutein (β-ε-dihydroxy-carotene), providing genetic evidence that LUT1 must be also active in the hydroxylation of the β-ring of α-carotene [12]. Introduction of the *lut1 *mutation in the *chy1chy2lut5 *triple mutant completely abolished xanthophyll biosynthesis, indicating that CHY1, CHY2, LUT1 and LUT5 constitute the full complement of carotenoid hydroxylases in Arabidopsis [14]. A number of studies [11-14] have shown a functional redundancy amongst the carotene hydroxylase enzymes. However, the extent of recovery in xanthophyll content by mutants carrying a single carotene hydroxylase activity gave insights on the substrate preference of residual activity. As an example, trace amounts of β-β-xanthophylls, present in the *chy1chy2lut5 *triple mutant and abolished by the introduction of the *lut1 *mutation [14], led to the conclusion that LUT1 has a low level of activity toward the β-rings of β-carotene. Overall, *in vivo *analysis clearly showed that CHY1 and CHY2 are most active in β-carotene hydroxylation, while LUT1 and LUT5 enzymes catalyze preferentially the biosynthesis of α-xanthophylls [14]. Here we show that, in the absence of α-carotene (whose synthesis is prevented by the *lut2 *mutation), LUT1 shows a major β-carotene hydroxylase activity, leading to the accumulation of substantial amounts of β-β-xanthophylls. Indeed, when considering the moles of hydroxylated β-rings accumulated in *chy1chy2lut5 *and *chy1chy2lut2lut5 *mutants, total amount is maintained upon introduction of the *lut2 *mutation into the *chy1chy1lut5 *background: ~0.125 μmol of hydroxylated β-rings/gFW in *chy1chy2lut5 vs*. ~0.118 μmol/gFW in *chy1chy2lut2lut5 *(Table 2). However, the total amount of xanthophylls per fresh weight is reduced significantly in *chy1chy2lut2lut5 *(-52%, see Table 2), while the carotene/xanthophyll ratio is increased (+57%). The most likely scenario is that LUT1 has a higher affinity towards ε-rings *vs*. β-rings and that, when α-carotene is available, LUT1 performs mainly ε-ring hydroxylation; however, since no ε-ring-substrates are available in the *chy1chy2lut2lut5*, the β-ring substrates are processed, thus bringing out this minor activity of the enzyme. Conversely, in the absence of the main hydroxylases for β-rings CHY1, CHY2 and LUT5, all β-rings become available for LUT1 activity in *chy1chy2lut2lut5*. A consequence of LUT1 operation on a less preferred substrate (i.e., β-carotene over α-carotene) is its reduced overall catalytic rate that limits the level of xanthophylls accumulated in the *chy1chy2lut2lut5 *genotype. It is worth noting that the β-hydroxylase activity does not discriminate between β-rings of α- and β-carotene; indeed, the total amount of hydroxylated β-rings per fresh weight is the same in both *chy1chy2lut5 *and *chy1chy2lut2lut5*. This evidence updates our knowledge on the molecular details of carotenoid hydroxylases, with respect to the view that LUT1 has only a low level of *in vivo *hydroxylase activity toward the β-carotene and a stronger activity towards α-carotene [14].

The concentration of β-carotene increased in the quadruple *chy1chy2lut2lut5 *mutant to a level higher than in the wild type (Table 2). This is the result of the combination of several factors: first, the *lut2 *mutation redirects the flux in the pathway towards the β-β-branch; this mutation alone is sufficient to cause a slight increase in β-carotene (Table 2). Moreover, the reduced hydroxylase activity due to the *chy1, chy2 and lut5 *mutations, reduces the rate of β-carotene processing into downstream xanthophylls (Figure 1E), favoring its accumulation. The almost 2-fold increase of the *LUT1 *transcript in the quadruple mutant with respect to the wild type (Figure 2) is insufficient to fully compensate for the disappearance of the CHY1, CHY2 and LUT5 hydroxylases, likely due to the low catalytic efficiency of LUT1 for β-rings.

### Reduced xanthophyll content negatively affects energy-dependent quenching qE and photoprotection

The excess energy dissipation into heat (NPQ) is strongly depleted in the *chy1chy2lut2lut5 *mutant, its amplitude being close to zero (Figure 3). Since the level of PsbS, the pH sensitive trigger for NPQ [30] is similar to wild-type, this effect can be attributed to the low level of the interacting partners of PsbS where the actual quenching occurs, i.e. the Lhcb proteins [31-34], and to the lack of lutein which limit NPQ [16,35]. All together these results support the correlation between xanthophyll content and amplitude of qE, previously indicated on the basis of antisense inhibition of beta hydroxylation [36]. However, the high photosensitivity of *chy1chy2lut2lut5 *plants is likely to be caused by the failure of additional photoprotection mechanisms, since the *npq4 *mutant, although depleted in NPQ, only showed minor increase in photosensitivity [30].

The fast leaf chlorophyll bleaching (Figure 4A) and high levels of lipid peroxidation (Figure 4B) in *chy1chy2lut2lut5 *with respect to wild-type, *lut2 and chy1chy2lut5 *are likely due to the strong depletion in Lhcb proteins (Figure 6B). Xanthophylls are needed for folding of Lhc proteins *in vitro *[37], thus it is not surprising that a strong decrease in their availability leads to a decreased content in LHC. However, only Lhcb proteins are affected, while Lhca proteins are maintained with the same stoichiometry with respect to PSI reaction center, as shown by the identical migration rate of PSI-LHCI supercomplexes in sucrose gradients and native gels (Figure 5B) and by quantitative immunotitration of Lhca versus PsaA content (Figure 6B). As a result of the higher stability of Lhca *vs*. Lhcb proteins, PSI antenna function is maintained, while PSII antenna function is impaired. We conclude that *in vivo *carotenes cannot replace xanthophylls in stabilizing Lhcb proteins, thus leading to their strong reduction. Furthermore, this implies that functional Lhcb proteins are essential for photoprotection, in agreement with a previous report [38]. Extreme reduction in Lhc proteins is obtained with the *ch1 *mutation in Arabidopsis, that impairs Chl *b *synthesis and prevents assembly of functional LHC [39]. Although this mutant undergoes severe photoinhibition in high light, it can grow on soil and is not photoinhibited in moderate light [7]. Thus *ch1*, with a PSII antenna size even smaller than that of *chy1chy2lut2lut5*, can survive in the absence of a reduced carbon source, a lethal condition for *chy1chy2lut2lut5*. We conclude that reduced LHC content, although likely contributing to sensitivity, cannot be the only reason for the extreme phenotype of *chy1chy2lut2lut5*.

### Limitation in total xanthophyll availability affects light-harvesting complex content and PSI/PSII ratio

The analysis of the pigment-protein complexes in the *chy1chy2lut2lut5 *mutant shows that Lhcb proteins are strongly decreased with respect to PSII, while Lhca assembly into PSI-LHCI are much less, or not at all, affected (Figures 5, 6). A reduction in Lhcb proteins is also observed in the *chy1chy2lut5 *mutant, albeit to a lesser extent than in *chy1chy2lut2lut5 *[6]. This effect is likely due to the incapacity of Lhcb proteins to fold in the absence of xanthophylls [37], while Lhca proteins can also bind small amounts of β-carotene [40,41]. Instead, there is no evident reason for the 5-fold decrease in PSI/PSII ratio (Figure 7): since Lhca proteins are maintained with the same stoichiometry with respect to PSI reaction center and thus likely contribute to the complex stability (Figures 5 and 6B), such a strong decrease of PSI is not expected. The dependence of PSI/PSII ratio on the xanthophyll/carotenoid ratio of different genotypes contrasts with the fact that both PSI core and PSII core complexes bind β-carotene [42] which is fully available in *chy1chy2lut2lut5*, as well as in the other genotypes carrying mutations in xanthophyll biosynthesis (Figure 7).

Alternatively, it can be hypothesized that PSI level might be limited by the amount of LHCI available. However, three lines of evidence are against the hypothesis that PSI depletion is a secondary effect of a limitation in LHCI:

1) Several Arabidopsis mutants showing a strong depletion of LHCI, including Lhca antisense lines [43] or the *ch1 *mutant [7,44] still accumulate a functional PSI core complex in moderate light [38].

2) P700^+ ^is not such a strong oxidizer as P680^+^, therefore photooxidative damages to PSI require very strong irradiance [45]. We have grown *chy1chy2lut2lut5 *and other genotypes (Figure 7) under moderate light, a condition that did not affect PSI activity in *ch1 *mutants [38]. Thus it is unlikely that a fraction of assembled PSI core complexes are destroyed due to lack of the LHCI moiety.

3) The capacity of LHCI to fold by binding both xanthophylls and β-carotene [41] makes these subunits less limited in their possibility to fold into pigment-protein complexes than Lhcb proteins.

We conclude that a tight correlation exists in plant thylakoids between PSI accumulation and xanthophyll availability which is not due to either direct stabilization of the complex by xanthophylls or by photoxidative stress. PSI and PSII core complex steady state level could be limited by chlorophyll availability, while a co-regulation of chlorophyll and carotenoid accumulation has been reported [46]. Nevertheless, the reduced amount of Chls in carotenoid biosynthesis mutants appears to be mainly due to de-stabilization of the carotenoid/chlorophyll-binding proteins [47]. Analysis of chlorophyll biosynthesis mutants [48] showed that PSI accumulation is less reduced than PSII accumulation, suggesting that the strong effect on PSI we observed in *chy1chy2lut2lut5 *is not due to limitation in Chls supply. The effect of norflurazon treatment, which shows a preferential effect on PSII activity [49], further suggests that the phenotype we observe is specific for PSI core and is specifically caused by xanthophyll depletion. The lack of xanthophylls in PSI core, however, suggests this specific effect must be indirect. One possibility is that xanthophylls, or their metabolites, control either PSI synthesis or degradation. A number of factors are involved in the synthesis of PSI and PSII subunits, either bound to the thylakoid membrane or soluble in the chloroplast stroma, that could be considered as tentative targets of regulation, including *srp *and *ftsy *[50,51] or *ATAB2 *protein [52]. Alternatively, carotenoid catabolites with regulatory roles [53] could be responsible for this effect. However, while the identification of the mechanisms underlying the down-regulation of PSI synthesis under limiting xanthophylls is beyond the scope of this manuscript, it is interesting to consider the implications that such a regulation would have on the function of the photosynthetic apparatus:

a) the regulation of xanthophyll/carotene level in high-light would reflect into a modulation of PSI level [54-56], thus alleviating PQ over-reduction and protecting from photoinhibition;

b) while PSII reaction centers are subjected to rapid turn-over and their level readily adjusted to environmental conditions, PSI is much more stable, thus requiring specific mechanisms for its down-regulation in limiting light. Lhcbs bind large amounts of xanthophylls and are strongly regulated depending on light intensity. Coupling PSI to xanthophyll levels would provide a mechanism for coordinated regulation of PSII antenna size and PSI/PSII ratio, a phenomenon observed in many species [57].

## Conclusions

One of the most noticeable results of recent work on the plant carotenoid biosynthesis pathway is the high level of redundancy in carotene hydroxylation, which is found to be catalyzed by 4 different enzymes. Here we show that the LUT1 protein, previously reported to act in α-carotene hydroxylation, has a major β-carotene hydroxylation activity, which is evidenced in the α-carotene-less genetic background of the *chy1chy2lut2lut5*. Surprisingly, in this mutant LHCI proteins are maintained with the same stoichiometry with respect to PSI reaction center. Unexpectedly, in spite of its correct folding, PSI reaction center is drastically reduced in *chy1chy2lut2lut5 *with respect to wild type, a condition that cannot be explained by a limitation in the availability of its LHCI moiety. Upon analysis of genotypes having different xanthophyll/carotenoid ratios, we show that xanthophyll availability correlates with PSI/PSII ratio within a wide range. The molecular mechanism(s) underlying regulation of both PSII antenna size and PSI/PSII ratio, alleviating PQ over-reduction during acclimation to excess light conditions, are being investigated.

## Methods

### Plant material and growth conditions

T-DNA insertion mutants were identified in the Syngenta and Salk collections. The knock-out lines mentioned in the article can be obtained from the NASC under the stock numbers N862308 (CHY1), N845663 (CHY2), N629724 (LUT1), N505018 (LUT2), N616660 (LUT5). Double and triple mutants were obtained as described [6]. To generate the quadruple mutant *chy1chy2lut2lut5*, the triple mutant *chy1chy2lut2 *and the single mutant *lut5 *were crossed, and F1 seeds were grown and self-fertilized to obtain the F2 generation. The genotype of the F2 individual seeds was checked by PCR using gene-specific and T-DNA primers [12]. We used two different parental genotypes for selection of the quadruple gene knockout (*chy1chy1chy2CHY2lut2lut2lut5lut5 *and *chy1chy1chy2chy2lut2lut2lut5LUT5*) identified by PCR from a segregating F_2 _population of a *chy1chy1chy2chy2lut2lut2LUT5LUT5 *x *CHY1CHY1CHY2CHY2LUT2LUT2lut5lut5 *cross. Progeny from each quadruple mutant parent genotype were analyzed on Petri plates containing 0.5× MS medium, 3.0% sucrose and 0.9% agar under a photoperiod of 16 h light (30 μmol photons m^-2 ^s^-1^). The genotypes of putative quadruple mutants were confirmed by PCR [12].

### In vivo fluorescence and NPQ measurements

Non-photochemical quenching of chlorophyll fluorescence (NPQ), its components qE and qI, and PSII yield (Φ_PSII_) was measured on whole leaves at RT (room temperature, 22°C) with a PAM 101 fluorometer (Walz, Germany). Leaves were given either 7 or 20 min of illumination in saturating CO_2_, and 15 min of dark-relaxation. Parameters were calculated during steady state photosynthesis according to [58].

### LC-MS analysis of leaf pigments

Chlorophyll/carotenoid extraction, LC separation and photodiode array were performed as previously described with slight modifications (Fraser *et *al., 2000). Briefly, 2-3 mg of ground lyophilized leaf powder were extracted with chloroform (spiked with 100 mg/l α-tocopherol acetate as internal standard) and methanol (2:1 by volume), 1 volume of 50 mM Tris buffer (pH 7.5, containing 1 M NaCl) was added and samples were kept 20 min on ice. After centrifugation (15,000 g for 10 min at 4°C), the organic hypophase was removed and the aqueous phase was re-extracted with spiked chloroform (2 by volume). Combined organic phases were then dried by speedvac and resuspended in 100 μl of ethyl acetate. For each genotype, at least four independent extractions were performed. LC-MS analyses were carried out using a Discovery LTQ-Orbitrap mass spectrometry system (Thermo Fischer Scientific) operating in negative mode-atmospheric pressure chemical ionization (APCI), coupled to an Accela U-HPLC system (Thermo Fischer Scientific, Waltham, MA). LC separations were performed using a C30 reverse-phase column (250 × 4.6 mm) purchased from YMC (YMC Europe GmbH, Schermbeck, Germany). The mobile phases used were methanol (A), water/methanol (20/80 by volume), containing 0.2% ammonium acetate (B), and tert-methyl butyl ether (C). The gradient was: 95%A:5%B for six minutes, followed by 80%A:5%B:15%C for 14 min and by a linear gradient to 30%A:5%B:65%C over 16 min. Detection was performed continuously from 220 to 700 nm with an online Accela Surveyor photodiode array detector (PDA, Thermo Fischer Scientific, Waltham, MA). All solvents used were LC-MS grade quality (CHROMASOLV^® ^from Sigma-Aldrich). Carotenoids were quantified on the basis of the internal standard amounts, obtained by through comparison with peak areas of known amounts of external standard LC-MS runs; data were then normalized on spectrophotometric chlorophyll contents. For APCI-MS ionization of xanthophylls (0-14 min of LC-MS run), nitrogen was used as sheath and auxiliary gas which were set to 25 and 5 units, respectively while the vaporizer temperature was 350°C, the capillary temperature was 250°C, the discharge current was set to 6.5 μA, the capillary voltage and tube lens settings were -2050 V and -77 V, respectively. APCI-MS ionization of carotenes (14-30 min of LC-MS runs) was performed with the following parameters: 40 and 10 unites of, respectively, nitrogen sheath and auxiliary gas; 250°C for vaporizer and capillary temperatures, 5.0 μA as discharge current, -30 and -110 as, respectively, capillary voltage and tube lens settings. Identification was performed by through comparison of chromatographic and spectral properties of authentic standards and reference spectra (Britton *et *al., 2004), and on the basis of the m/z accurate masses, as reported on Pubchem database http://pubchem.ncbi.nlm.nih.gov/ for monoisotopic masses identification, or on Metabolomics Fiehn Lab Mass Spectrometry Adduct Calculator http://fiehnlab.ucdavis.edu/staff/kind/Metabolomics/MS-Adduct-Calculator/ in case of adduct ion detection.

### Thylakoid isolation

Thylakoids were isolated from leaves as previously described [59]. Membranes (70 μg of chlorophylls) were washed twice with 5 mM EDTA, 20 mM Hepes pH 7.8, then solubilized in 150 μl of 0.8% α-Dodecyl-maltoside (α-DM), 10 mM HEPES pH 7.5. Solubilized samples were then fractionated by ultracentrifugation (5.5 h at 60,000 rpm, 4°C) in a 0.1-1 M sucrose gradient containing 0.06% α-DM.

### Gel electrophoresis

SDS-PAGE analysis was performed with either the Tris-Tricine or the Tris-Glycine buffer systems as previously described [60]. Non-denaturing Deriphat-PAGE was performed as described by [61]. For immunotitration, thylakoid samples corresponding to 0.05, 0.1, 0.25 and 0.5 μg of chlorophyll were loaded for each sample and electroblotted on nitrocellulose membranes. Filters were incubated with specific antibodies and were detected with alkaline phosphatase-conjugated antibody [62]. Gel images were quantified using GelPro 3.2 (Bio-Rad). Samples compared were loaded in the same slab gel.

### Spectroscopy

Spectra were recorded on samples in 10 mM HEPES pH 7.5, 0.06% α-DM, 0.2 M sucrose, using an SLM-Aminco DW-2000 spectrophotometer at RT.

### Determination of the sensitivity to photooxidative stress

Photooxidative stress was induced in detached leaves by a strong light treatment at low temperature. Detached leaves on wet filter paper were exposed to high light (900 μmol photons m^-2 ^s^-1^, 5°C) for 3.5 h, then immediately frozen in liquid nitrogen. Photooxidative stress was assessed by measuring malondialdehyde (MDA) formation [63]; the thiobarbituric acid adduct MDA-(TBA)_2 _was quantified by HPLC [21].

### Real-time PCR

Total RNA was isolated from frozen tissue and analyzed through Real Time RT-PCR using previously published methods [64]. Three independent RNA extractions (from three pools of at least ten plants each) and three cDNAs (one for each RNA extraction) were used for the analyses; first strand cDNA was synthesized from 0.5 μg of RNA in 20 μl with oligo-dT(16) and Superscript II (Invitrogen). Real Time PCR was performed using an ABI PRISM 7000 instrument and the SYBR Green Master Mix kit (Applera). Standard dilution curves were performed for each gene fragment and all data were normalized for the β-TUBULIN transcript and for wild-type expression levels. Primers for Real Time experiments (Additional file 1: Table S1) were designed using the Primer Express v2.0 software and validated with the Amplify v3.1 software.

### Statistics

Significance analyses were performed using an analysis of variance with a pair-wise multiple comparison procedure in Origin. Error bars represent the standard deviation.

## Abbreviations

PSI and PSII: Photosystem I and II: respectively; α-DM: *n*-dodecyl-α-D-maltoside; Car: carotenoids; Chl *a *and *b*: chlorophyll *a *and b: respectively; F_v_/F_m_: maximal PSII photochemical efficiency; gFW: gram of fresh weight; HL: high-light; HPLC-DAD-MS: high pressure liquid chromatography - diode array detector - mass spectrometry; Lhca and Lhcb: light-harvesting complexes of PSI and PSII: respectively; LHCI: antenna complex of photosystem I; LHCII: major light-harvesting complex of PSII; MDA: malondialdehyde; NPQ: non-photochemical quenching; PQ: plastoquinone; qE: ΔpH-dependent component of NPQ; qI: photoinhibition quenching; qP: photochemical quenching; ROS: reactive oxygen species; RT: room temperature; TBA: thiobarbituric acid; Φ_PSII_: efficiency of PSII photochemistry.

## Authors' contributions

AF performed identification and isolation of all the genotypes used, carried out the molecular genetic studies and drafted the manuscript; LD and SC carried out the biochemical and photosynthetic characterization of plants under control and photoxidative conditions, performed western-blot analysis and drafted the manuscript; GD performed mass-spectrometry analysis; GG and RB conceived the study, participated in its design and coordination and edited the manuscript. All authors read and approved the final manuscript.

## Supplementary Material

Additional file 1 **Figure S1**. Genomic structure of the different mutants utilized. Figure S2. PCR confirmation of the different mutants. Figure S3. Isolation and characterization of the pigment-protein complexes from wild-type and *chy1chy2lut2lut5 *thylakoid membrane. Figure S4. Distribution of the PSI core (**A**), PSII core (**B**) and LHCII (**B**) amount per fresh weight *vs*. the relative content of xanthophylls on thylakoids. Table S1. Sequences of oligonucleotides used for RT-PCR measurement of transcripts. Table S2. LC-DAD-MS analysis of wild-type and mutant Arabidopsis leaves. Click here for file
