# Supplementary material for: A quadruple mutant of Arabidopsis reveals a β-carotene hydroxylation activity for LUT1/CYP97C1 and a regulatory role of xanthophylls on determination of the PSI/PSII ratio
Source: BMC Plant Biol. 2012 Apr 18;12:50. doi: 10.1186/1471-2229-12-50 (PMC3349566; doi:10.1186/1471-2229-12-50)
Supplement: Additional file 1 — Figure S1. Genomic structure of the different mutants utilized. Figure S2. PCR confirmation of the different mutants. Figure S3. Isolation and characterization of the pigment-protein complexes from wild-type and chy1chy2lut2lut5 thylakoid membrane. Figure S4. Distribution of the PSI core (A), PSII core (B) and LHCII (B) amount per fresh weight vs. the relative content of xanthophylls on thylakoids. Table S1. Sequences of oligonucleotides used for RT-PCR measurement of transcripts. Table S2. LC-DAD-MS analysis of wild-type and mutant Arabidopsis leaves. [file 1471-2229-12-50-S1.DOC]

**Additional results**

**Additional Figure 1**

**
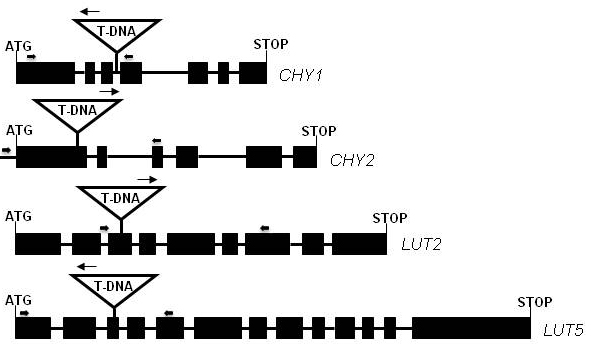
**

**Additional Figure 1. Genomic structure of the different mutants utilized.** The T-DNA insertions are indicated as triangles. Thick arrows represent gene-specific primers, thin arrows represent the T-DNA-specific primer.

**Additional Figure 2.**

**
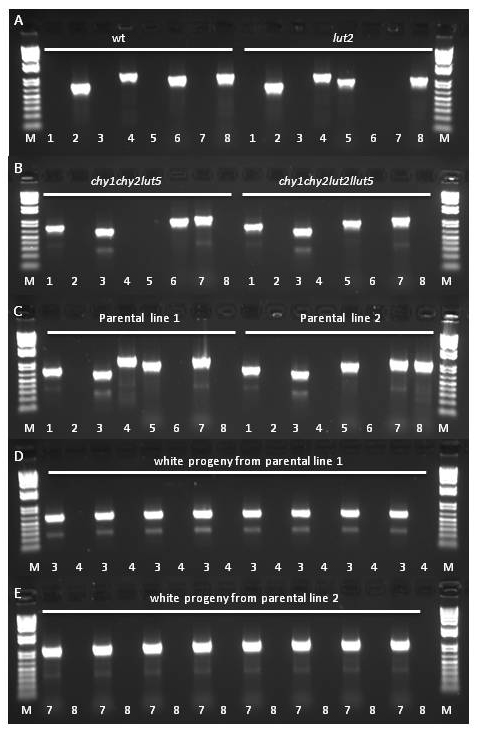
**

**Additional Figure 2. PCR confirmation of the different mutants.** (A) Wild-type and *lut2* mutant. (B) *chy1chy2lut5* (triple) and *chy1chy2lut2lut5* (quadruple) mutants. (C) Parental line 1 (*chy1lut2lut5* triple mutant, heterozygous for the *chy2* mutation) and parental line 2 (*chy1chy2lut2* triple mutant, heterozygous for the *lut5* mutation) for the *chy1chy2lut2lut5* quadruple mutant. (D) Analysis of the *CHY2* gene in eight white seedlings deriving from parental line 1; all seedlings are *chy2*/*chy2.* (E) Analysis of the *LUT5* gene in eight white seedlings deriving from parental line 2; all seedlings are *lut5/lut5.* M: molecular weight marker. 1: *CHY1*:T-DNA amplicon. 2: *CHY1* gene-specific amplicon. 3: *CHY2*: T-DNA amplicon. 4: *CHY2* gene-specific amplicon. 5: *LUT2*: T-DNA amplicon. 6: *LUT2* gene-specific amplicon. 7: *LUT5*:T-DNA amplicon. 8: *LUT5* gene-specific amplicon.

**Additional Figure 3**

**Additional Figure 3. Isolation and characterization of the pigment-protein complexes from wild-type and *chy1chy2lut2lut5* thylakoid membrane.** Absorption spectra of the monomeric Lhcb (band 2, panel A), PSII core complex (band 5, panel B) and PSI-LHCI complexes (band 6, panel C) isolated by sucrose gradient ultracentrifugation from wild-type and *chy1chy2lut2lut5* thylakoids (see Figure 5B). The spectra are normalized to the maximum in the red region. The Chl *b* content, which is proportional to the Lhc antenna content, is obtained from the amplitude of the absorption components at 470 and 650 nm.

**Additional Figure 4.**

**
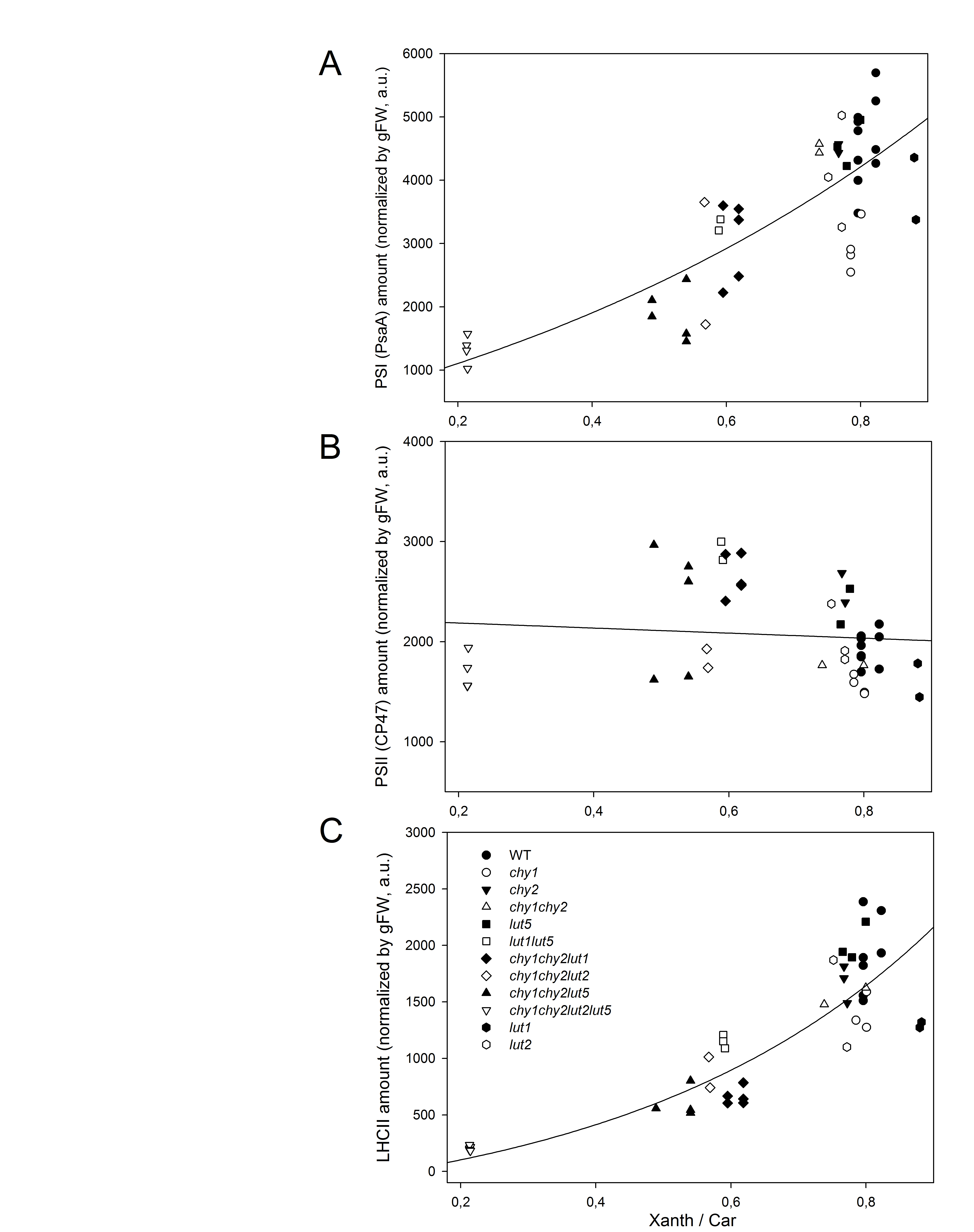
**

**Additional Figure 4. Distribution of the PSI core (A), PSII core (B) and LHCII (B) content per gram of fresh weight *vs.* the xanthophyll/carotenoid ratio.** Protein amounts were measured for wild-type and 11 xanthophyll biosynthesis mutants plants previously described (Fior*e et a*l., 2006; Dall'Ost*o et a*l., 2007b; present ms). Protein amounts per fresh weight were determined on leaf extracts by quantitative western blot, while xanthophyll/carotenoid ratios were quantified by HPLC. gFW, gram of fresh weight. R^2^_PSI amount_ = 0.65; R^2^_PSII amount_ = 0.01; R^2^_LHCII amount_ = 0.73.

**Additional Tables**

**Additional Table 1**

| **Genes** | **Oligonucleotides** |
| --- | --- |
| CHY1 forward | GCTCTCTCTGTTGGTGCTGCT |
| CHY1 reverse | AGCCCATCTTGCCCAGAAT |
| CHY2 forward | TTTGCTCTTTCCGTTGGTGC |
| CHY2 reverse | CCATCTTGCCCAAAACTCCAT |
| LUT1 forward | ACATAGATGGCGCAATCCCT |
| LUT1 reverse | CTACACATTTTCTAGGCCCTCCA |
| LUT2 forward | GTATTGAGCATGTTTGGAG |
| LUT2 reverse | TCGTAAGTCTTTAGAATTCG |
| LUT5 forward | CGGCGGCAGTATTAACATGG |
| LUT5 reverse | CACTTGGTTCCGTTGTTAAAAGG |
| TUB forward | GGTGTTCCCTTCACCAAAGGT |
| TUB reverse | GCGTTGTAAGGCTCCACCAC |

**Additional Table 1.** Sequences of oligonucleotides used for RT-PCR measurement of transcripts. TUB, tubulin.

**Additional Table 2**

**Additional Table 2.** LC-DAD-MS analysis of wild-type and mutant Arabidopsis leaves. Retention time (RT), maximum absorbance wavelenghts, chemical formula and theoretical (as reported on Pubchem:http://pubchem.ncbi.nlm.nih.gov/) and experimental accurate masses for each metabolite are reported. Experimental formula was obtained using the Metabolomics Fiehn Lab Mass Spectrometry Adduct Calculator (http://fiehnlab.ucdavis.edu/staff/kind/Metabolomics/MS-Adduct-Calculator/). M: monoisotopic mass; Hac: acetic acid. For more details, see Methods.
